# Older adults with sarcopenia have distinct skeletal muscle phosphodiester, phosphocreatine, and phospholipid profiles

**DOI:** 10.1111/acel.13135

**Published:** 2020-05-28

**Authors:** James Matthew Hinkley, Heather H. Cornnell, Robert A. Standley, Emily Y. Chen, Niven R. Narain, Bennett P. Greenwood, Valerie Bussberg, Vladimir V. Tolstikov, Michael A. Kiebish, Fanchao Yi, Rick B. Vega, Bret H. Goodpaster, Paul M. Coen

**Affiliations:** ^1^ AdventHealth Translational Research Institute Orlando FL USA; ^2^ BERG Health Framingham MA USA

**Keywords:** aging, muscle volume, peak power, phosphatidylcholine, phosphatidylethanolamine, phosphodiester

## Abstract

The loss of skeletal muscle mass and function with age (sarcopenia) is a critical healthcare challenge for older adults. 31‐phosphorus magnetic resonance spectroscopy (^31^P‐MRS) is a powerful tool used to evaluate phosphorus metabolite levels in muscle. Here, we sought to determine which phosphorus metabolites were linked with reduced muscle mass and function in older adults. This investigation was conducted across two separate studies. Resting phosphorus metabolites in skeletal muscle were examined by ^31^P‐MRS. In the first study, fifty‐five older adults with obesity were enrolled and we found that resting phosphocreatine (PCr) was positively associated with muscle volume and knee extensor peak power, while a phosphodiester peak (PDE2) was negatively related to these variables. In the second study, we examined well‐phenotyped older adults that were classified as nonsarcopenic or sarcopenic based on sex‐specific criteria described by the European Working Group on Sarcopenia in Older People. PCr content was lower in muscle from older adults with sarcopenia compared to controls, while PDE2 was elevated. Percutaneous biopsy specimens of the vastus lateralis were obtained for metabolomic and lipidomic analyses. Lower PCr was related to higher muscle creatine. PDE2 was associated with glycerol‐phosphoethanolamine levels, a putative marker of phospholipid membrane damage. Lipidomic analyses revealed that the major phospholipids, (phosphatidylcholine, phosphatidylethanolamine, and phosphatidylglycerol) were elevated in sarcopenic muscle and were inversely related to muscle volume and peak power. These data suggest phosphorus metabolites and phospholipids are associated with the loss of skeletal muscle mass and function in older adults.

## Graphical abstract

Using in vivo measures and biochemical analyses of muscle biospecimens, we observed that sarcopenia invokes a distinct phosphorus metabolite profile which are associated with the loss of muscle mass and function in older adults. Specifically, reduced phosphocreatine levels were related with lower muscle mass and function. Additionally, elevations in markers of cellular membrane dysfunction (PDE2, glycerol‐phosphoethanolamine, and phospholipids) were associated with the sarcopenic phenotype.

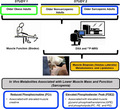

## 1 INTRODUCTION

The population of older adults will more than double to 98 million by 2050 and represents a major healthcare challenge (Olshansky, Goldman, Zheng, & Rowe, 2009). Older adults have a higher risk of developing many chronic diseases and are at increased risk of falls, fractures, and mobility disability. A primary determinant of reduced mobility in older adults is low muscle mass and function, also known as sarcopenia. Unfortunately, despite the clinical relevance, the cellular mechanisms that contribute to sarcopenia are unclear, thus impeding the discovery of potential therapeutic options to treat the condition.

Resting phosphorus metabolites play a significant role in skeletal muscle health by influencing various physiological processes, including providing high‐energy phosphates for contractile activity or structural stability to phospholipid‐dense cellular membranes. 31‐phosphorus magnetic resonance spectroscopy (^31^P‐MRS) is an important tool to assess in vivo phosphorus metabolites and has been used to study skeletal muscle in aging and obesity. Dynamic changes in the phosphorus metabolite profile following an acute bout of muscle contraction provide a surrogate for mitochondrial function, and previous reports have shown impairments with obesity, type 2 diabetes, and aging (McCully et al., 1991). Though not as well studied, recent evidence has revealed that resting in vivo phosphorus metabolite levels may relate to whole‐body and muscle‐specific phenotypes. Previous reports have shown dysregulation of resting in vivo phosphorus metabolites under pathological conditions, including obesity, type 2 diabetes, spinal cord injury, and muscular dystrophy, and appear to be linked to clinical measures of glycemic control and body composition (Hooijmans et al., 2017; McCully, Mulcahy, Ryan, & Zhao, 2011; Ripley et al., 2018; Szendroedi et al., 2011). Specifically, resting phosphocreatine (PCr) levels have been shown to be negatively associated with HbA1c and fasting glucose levels, while heightened levels are linked with elevated intracellular ATP levels (Ripley et al., 2018). Additionally, phosphodiester (PDE) content, a putative marker of skeletal muscle membrane damage, is elevated under conditions of lower muscle mass in spinal cord injury (McCully et al., 2011) and muscular dystrophy (Hooijmans et al., 2017). Furthermore, elevated PDE is associated with heightened HbA1c (Ripley et al., 2018; Szendroedi et al., 2011), reduced insulin sensitivity (Ripley et al., 2018; Szendroedi et al., 2011), increased body mass (Szendroedi et al., 2011), and elevated intramyocellular lipids (Ripley et al., 2018). Along with in vivo measures, biochemical analyses of muscle biospecimens have revealed an integral role of phosphorus metabolites in metabolic health. Of note, insulin sensitivity appears to be influenced by resting skeletal muscle phospholipid composition in athletes, obese adults, and diabetics (Newsom et al., 2016). While resting phosphorus metabolites appear to be linked to clinical endpoints that are critical for metabolic health, it is unclear how these metabolites relate to muscle mass and function in older adults.

The purpose of this study was to examine the relationship between resting phosphorus metabolites with skeletal muscle mass and function in older adults. We first identified which phosphorus metabolites were related to measures of skeletal muscle mass and function in a group of fifty‐five older adults. We next examined whether the in vivo metabolites associated with lower muscle mass and function were dysregulated with sarcopenia in a second cohort of well‐phenotyped sedentary older adults classified as nonsarcopenic and sarcopenic. We hypothesized that sarcopenic muscle invokes a distinct in vivo phosphorus metabolite profile which are associated with the loss of muscle mass and function in older adults.

## 2 RESULTS

### 2.1 Acquisition parameters to identify resting phosphorus metabolites using ^31^P‐MRS

**FIGURE 1:**
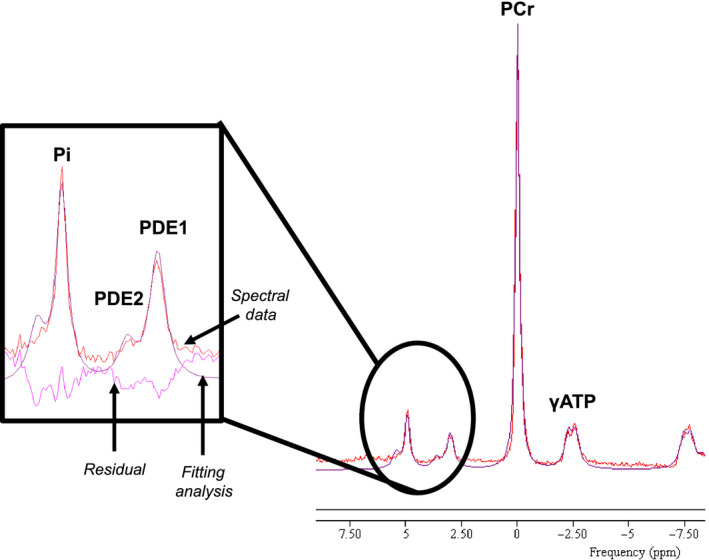
Representative ^31^P‐MRS spectra from thigh muscle of older adult participant. Trace was taken from older participant without sarcopenia (*Study 2*). The spectral data are shown in red, the smooth line overlaying the raw data (purple line) is the result of the fitting analysis, and the residual (pink line) is shown as the difference between the raw data and the fit. Inset: peaks that identify inorganic phosphate (Pi), and 2 phosphodiester (PDE) peaks (PDE1 and PDE2). Additionally, lines for spectral data, fitting analysis, and residual are highlighted

In the present study, we utilized a 3T Philips Achieva magnet to identify resting phosphorus metabolites in skeletal muscle by ^31^P‐MRS. To improve spectral resolution to quantify smaller peaks within the phosphorous spectra, the fully relaxed spectrum was obtained over a 12‐min total acquisition time, with data collected 48 times at a repetition time of 15 s. The quality of the fitting parameters was optimized and tested prior to use in this study, and all reported peaks had a coefficient of variance of 0.55% or less using our fitting parameters. By increasing the acquisition time, we were able to identify and quantify two phosphodiester peaks (PDE1 and PDE2) that were located approximately + 2.5 ppm from the PCr peak in the fully relaxed spectra. Representative fully relaxed spectra can be found in Figure 1.

### 2.2 In vivo PCr and PDE2 are associated with muscle mass and function in older adults with obesity

**FIGURE 2:**
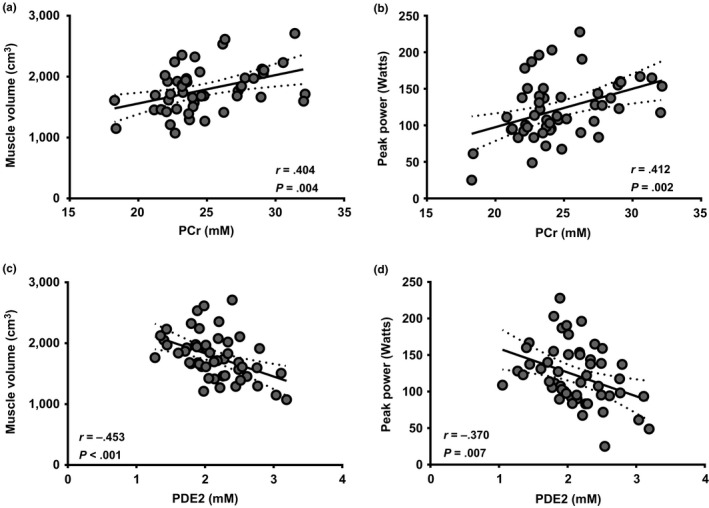
PCr and PDE2 are associated with whole‐muscle mass and function in a homogeneous group of older obese adults. ^31^P‐MRS was used to quantify in vivo PCr and PDE2 levels in older obese adults. Pearson correlations were then examined associations concentrations (in mM) of PCr (a–b) and PDE2 (c–d) with (a and c) muscle volume (cm^3^), and (b and d) peak power (Watts). *N* = 55. Best‐fit trend line (solid line) and 95% confidence intervals (dotted line) are included

Previous studies have linked resting phosphorus metabolites with metabolic health in humans (Hooijmans et al., 2017; McCully et al., 2011; Ripley et al., 2018; Szendroedi et al., 2011), yet, it is uncertain whether resting in vivo metabolites are associated with muscle mass and function in older adults. The goal for *Study 1* was to explore the covariance between muscle phenotype and resting in vivo phosphorus metabolites. To do this, older adults with obesity (*n* = 55 [12 males, 43 females]); BMI = 36.2 kg/m^2^ (range: 30.1–49.4 kg/m^2^); body weight = 97.3 kg (range: 74.4–136.4 kg) who were classified as type 2 diabetic (*n* = 18) or nondiabetic (*n* = 37) were examined. These participants, while all obese, had a wide range in muscle mass markers (skeletal muscle index [SMI]: 6.2–11.0 ALM/hr^2^; muscle volume: 1,073.1–2,709.4 cm^3^) as well as muscle functionality (peak power: 25.0–227.8 W). Despite other reports linking phosphorus metabolites with insulin sensitivity (Ripley et al., 2018; Szendroedi et al., 2011), we found that type 2 diabetes status did not influence the in vivo metabolic phenotype (data not shown), and therefore, all subjects were grouped for correlative analyses. Next, we examined relationships with body composition measurements and found that SMI, an index of muscle mass used to define sarcopenic status, was associated with two metabolites, PCr (*r* = .365; *p*‐value = .008) and PDE2 (*r* = −0.366; *p*‐value = .008). For a more precise assessment of skeletal muscle mass, we examined muscle volume by magnetic resonance imaging (MRI) and found that PCr was positively associated with muscle volume (Figure 2a) while PDE2 was negatively associated (Figure 2c). To determine whether these relationships extended to muscle function, we examined knee extensor power and found that peak power was positively related to PCr (Figure 2b) and negatively related to PDE2 (Figure 2d). Levels of Pi and PDE1 were not related to muscle mass or function (data not shown).

It has previously been shown that in vivo phosphorus metabolites are related to type 2 diabetes status and body/fat composition (Szendroedi et al., 2011). While fat mass was not related to in vivo phosphorus metabolites, region % fat was negatively related to PCr, while PDE2 was positively associated (Table S1). To further delineate the role of type 2 diabetes status and body/fat mass on the relationship between in vivo phosphorus metabolites and muscle mass and function, we performed multiple regression analyses. For muscle volume, we found that PCr had a greater influence than type 2 diabetes status (Beta: 0.387 [PCr] vs. 0.060 type 2 diabetes status), BMI (Beta: 0.384 [PCr] vs. −0.024 BMI), and fat mass (Beta: 0.370 [PCr] vs. −0.100 fat mass). Similarly, PDE2 had a greater influence on muscle volume than type 2 diabetes status (Beta: −0.473 [PDE2] vs. 0.072 type 2 diabetes status), BMI (Beta: −0.742 [PDE2] vs. −0.070 BMI), and fat mass (Beta: 0.461 [PDE2] vs. −0.128 [Fat Mass]). The results were similar for peak power. These data are the first evidence to link distinct in vivo metabolites (PCr and PDE2) to skeletal muscle mass and function in older adults.

### 2.3 Participant characteristics for Study 2

We next examined the relationships between PCr and PDE2 with muscle mass and function in a separate cohort of older adults with and without sarcopenia. We recruited subjects that were classified as nonsarcopenic or sarcopenic based on the sex‐specific clinical definition outlined by the 2010 European Working Group on Sarcopenia in Older People (EWGSOP; Cruz‐Jentoft et al., 2010). Participant characteristics can be found in Table 1. Per study design, sarcopenic participants had lower SMI and hand grip strength in comparison to nonsarcopenic controls. Additionally, muscle volume and peak power of the quadriceps muscles were lower with sarcopenia. When separated by gender, both females and males with sarcopenia presented lower muscle volume, with trends (*p* = .09) for lower SMI. Though not statistically significant, an imbalance in male/female ratio was observed between groups. Therefore, sex was considered a co‐variate in ANOVA analysis at baseline and in correlative analyses.

**Table 1:** Subject characteristics for *Study 2*

	Group			Females			Males		
	NonSarcopenic	Sarcopenic	*p*‐value	NonSarcopenic	Sarcopenic	*p*‐value	NonSarcopenic	Sarcopenic	*p*‐value
Age (years)	70.7 ± 1.2	70.9 ± 1.7	.9307	67.1 ± 0.9	71.6 ± 2.1	.1598	73.1 ± 1.5	69.0 ± 4.0	.2196
Sex (M/F)	10/7	2/5	.3707						
Weight (kg)	79.9 ± 3.4	65.1 ± 3.9	.0952	72.0 ± 3.9	59.4 ± 1.8	.1301	85.4 ± 4.4	79.1 ± 3.0	.4720
BMI (kg/m^2^)	27.9 ± 0.8	24.0 ± 0.8	.0471	27.5 ± 1.3	23.3 ± 0.7	.0667	28.1 ± 1.1	25.8 ± 1.9	.3626
SMI (ALM/h^2^)	7.6 ± 0.3	6.0 ± 0.3	.0095	6.5 ± 0.3	5.5 ± 0.1	.0860	8.3 ± 0.3	7.6 ± 0.1	.0860
Hand grip strength (kg)	32.3 ± 1.8	23.1 ± 2.1	.0161	26.4 ± 1.1	21.5 ± 2.2	.1519	36.5 ± 2.0	27.1 ± 5.0	.0828
Gait Speed (m/s)	1.03 ± 0.04	0.80 ± 0.05	.3052	1.07 ± 0.09	0.84 ± 0.05	.0452	0.99 ± 0.05	1.06 ± 0.02	.5923
Muscle Volume (cm^3^)	1899.7 ± 112.4	1,375.8 ± 151.5	.0021	1,401.6 ± 69.6	1,145.2 ± 35.1	.0260	2,248.4 ± 55.8	1952.3 ± 64.5	.0275
Peak Power (Watts)	139.3 ± 11.5	83.7 ± 10.9	.0343	107.7 ± 2.7	67.8 ± 4.6	.1301	161.4 ± 16.3	123.6 ± 6.8	.1812

Abbreviations: ALM/h^2^, appendicular lean mass per height^2^; BMI, body mass index; cm^3^, centimeters^3^; kg, kilograms; kg/m^2^, kilograms per meters^2^; m/sec, meters per second; SMI, skeletal muscle index.

### 2.4 PCr–Creatine axis in older adults with and without sarcopenia

**FIGURE 3:**
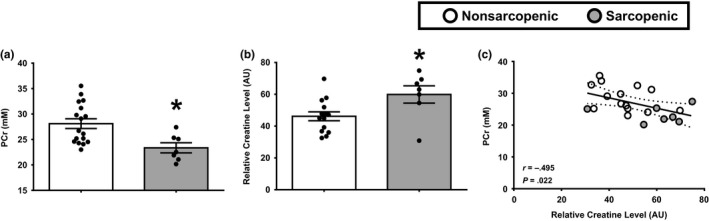
Lower in vivo phosphocreatine levels are associated with elevated creatine levels in sarcopenic skeletal muscle. (a) ^31^P‐MRS was used to quantify in vivo phosphocreatine (PCr) metabolites in the thigh muscle of older nonsarcopenic (white bars) and sarcopenic (gray bars) adults. (b) Quantitative analysis of skeletal muscle creatine levels was examined in muscle biospecimens from older nonsarcopenic and sarcopenic adults. *p ≤ .05 vs. nonsarcopenic. *N* = 7–16 participants per group. (c) Pearson correlations were first used to determine the relationship between in vivo concentrations of phosphocreatine (PCr, mM) and biochemical analysis of creatine levels. *N* = 21 participants total. White circles, nonsarcopenic adults; gray circle, sarcopenic adults. Best‐fit trend line (solid line) and 95% confidence intervals (dotted line) are included

Sarcopenic adults had reduced in vivo skeletal muscle levels of PCr in comparison to nonsarcopenic counterparts (Figure 3a). Similar to the findings in *Study 1*, PCr tended to be positively associated with muscle volume (Pearson *r* = .381, *p*‐value = .073) and peak power (Pearson *r* = .355, *p*‐value = .097) in sarcopenic and nonsarcopenic older adults. To understand potential factors that may influence alterations in resting PCr, we performed a metabolomic screen on muscle biopsy specimens obtained from participants in *Study 2*. Our analysis revealed a heightened level of creatine in sarcopenic muscle (Figure 3b). This is significant as PCr is replenished through the creatine kinase shuttle, which utilizes ATP generated by the mitochondria and creatine as substrates. In addition, muscle creatine levels were negatively associated with in vivo PCr content (Figure 3c).

### 2.5 Elevated membrane breakdown byproduct glycerol‐phosphoethanolamine (GPE), a biochemical readout of PDE2, in sarcopenic muscle

**FIGURE 4:**
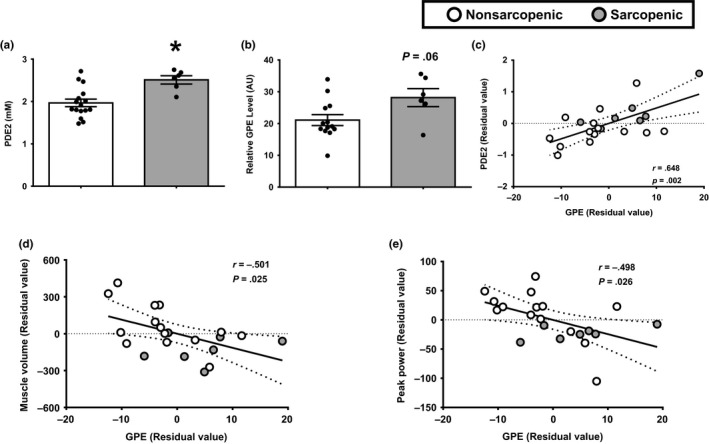
Increases in the metabolic constituent of PDE2, GPE, are related to lower muscle mass and function. (a) ^31^P‐MRS was used to quantify in vivo phosphocreatine (PCr) metabolites in the thigh muscle of older nonsarcopenic (white bars) and sarcopenic (gray bars) adults. (b) Quantitative analysis of skeletal muscle GPE levels was examined in muscle biospecimens from older nonsarcopenic and sarcopenic adults. *p ≤ .05 vs. nonsarcopenic. *N* = 7–16 participants per group. Pearson correlations were first used to determine the relationship between in vivo concentrations of PDE2 and GPE (c), as well as the relationship between skeletal muscle concentrations of GPE and (d) muscle volume and (e) peak power. *N* = 21 participants total. Data are presented as residual values, which were derived from a regression model taking gender into account. White circles, nonsarcopenic adults; gray circle, sarcopenic adults. Best‐fit trend line (solid line) and 95% confidence intervals (dotted line) are included

In *Study 2* participants, PDE2 was significantly elevated in older sarcopenic adults in comparison to nonsarcopenic counterparts (Figure 4a). In addition, PDE2 was negatively related to muscle volume (Pearson *r* = −.625, *p*‐value = .001) and a trend with peak power (Pearson *r* = −.334, *p*‐value = .12). Biochemically, the PDE2 signal in an MR spectra is thought to be due to GPE levels in the tissue and has been postulated to reflect phospholipid membrane breakdown or damage (Burt, 1985). Baseline comparisons revealed a trend for muscle GPE to be increased in muscle of older sarcopenic adults (Figure 4b). Additionally, we observed a positive relationship between PDE2 and GPE in skeletal muscle (Figure 4c). Similar to PDE2, the elevation in GPE was negatively associated with whole‐muscle mass and function (Figure 4d,e).

### 2.6 Major phospholipids are increased in sarcopenic muscle and relate to reduced muscle size and function

**FIGURE 5:**
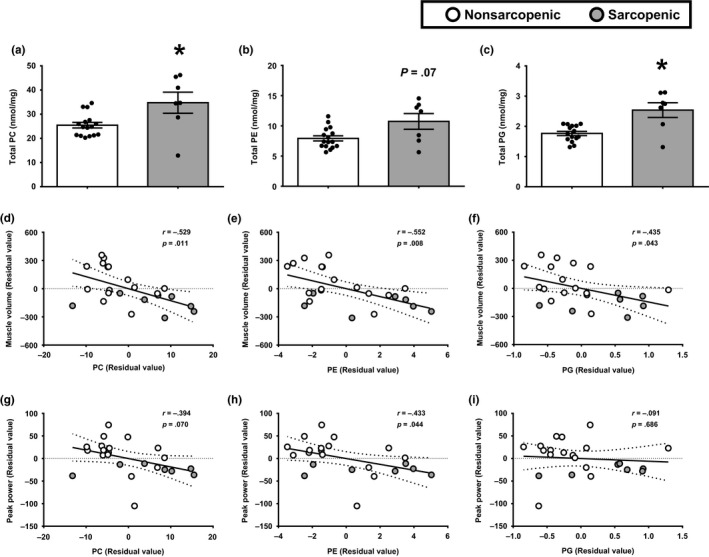
Elevated skeletal muscle phospholipids in sarcopenic muscle are associated with whole‐muscle mass and function in older adults with and without sarcopenia. Total content of phosphatidylcholine (PC) (a), phosphatidylethanolamine (PE) (b), and phosphatidylglycerol (PG) (c) were examined in skeletal muscle biopsy samples from nonsarcopenic (white bars) and sarcopenic (gray bars) participants. *p ≤ .05 vs. nonsarcopenic. *N* = 7–16 participants per group. Pearson correlations were used to examine associations with the total levels of PC (d and g), PE (e and h), and PG (f and i) with muscle volume (d–f), and peak power (g–i). *N* = 23. Data are presented as residual values, which were derived from a regression model taking gender into account. White circles, nonsarcopenic adults; gray circle, sarcopenic adults. Best‐fit trend line (solid line) and 95% confidence intervals (dotted line) are included

Based on the changes in the putative markers of membrane damage PDE2 and GPE and the implication for potential alterations in phospholipid membranes, we next examined the phospholipid profile in skeletal muscle. To do this, we employed a lipidomic approach to measure the major classes of phospholipids, with a focus on phosphatidylcholine (PC), phosphatidylethanolamine (PE), and phosphatidylglycerol (PG). We found that total PC, PE, and PG levels were increased in sarcopenic muscle (Figure 5a–c). The differences in phospholipid composition appear to be linked to the sarcopenic phenotype, as total PC (Figure 5d,g) and PE (Figure 5e,h) were negatively associated with muscle volume and peak power. Total PG was negatively associated with muscle volume (Figure 5f); however, PG levels were not related to peak power (Figure 5i). Taken together, these data indicate alterations in membrane phospholipid composition may be linked to impaired muscle mass and function in older adults with sarcopenia.

## 3 DISCUSSION

In the current study, we utilized ^31^P‐MRS to examine resting phosphorus metabolites in older adults. Our results revealed distinct phosphorus metabolites that are associated with lower muscle mass and function. Specifically, lower in vivo PCr in sarcopenic muscle was related to lower muscle mass and function in older adults. Additionally, we show that increased in vivo skeletal muscle PDE2 levels, a marker of impaired phospholipid membrane integrity, were associated with lower muscle mass and function in older adults with and without sarcopenia. Collectively, our data indicate phosphorus metabolites and phospholipids may be related to changes in muscle mass and function in older adults.

### 3.1 Muscle PCr is linked to skeletal muscle mass and function in older adults

Our results of lower muscle PCr levels in aging are in line with previous literature that examined the influence of age on skeletal muscle metabolite levels (McCully et al., 1991; Möller, Bergström, Fürst, & Hellström, 1980; Smith et al., 1998). A reduction in muscle PCr in older adults is not consistent in all studies, with other groups showing no difference (Kent‐Braun & Ng, 2000) or even an increase (Conley, Jubrias, & Esselman, 2000) in older adults. The discrepancy between studies could be due to the muscle examined. In comparison to the current study in which we examined the thigh muscles, Kent‐Braun and Ng observed no differences in PCr levels in the tibialis anterior (TA) muscle between young and older participants (Kent‐Braun & Ng, 2000). However, in contrast to previous work (McCully et al., 1991), in vivo oxidative capacity was higher in the TA of older adults in comparison to younger counterparts despite lower physical function and minutes of moderate‐to‐vigorous physical activity (Tevald, Foulis, & Kent, 2014). This would suggest additional factors (i.e., gait biomechanics) may influence the resting metabolic profile in muscle with age. Furthermore, the methodology used to quantify skeletal muscle metabolites may result in discrepant findings. Conley et al showed elevated PCr in skeletal muscle of older adults using ^31^P‐MRS (Conley et al., 2000), yet, using biochemical analyses in muscle biospecimens from the same subjects, the authors revealed similar levels between young and old participants. Additionally, when the biochemical values were compared to previous results in younger adults (Harris, Hultman, & Nordesjö, 1974), PCr levels appeared to be lower in the older group, which is similar to findings in the current study.

To further probe potential causes of the reduction in PCr levels, we performed a metabolomic screen and observed elevated creatine content in muscle biospecimens from older sarcopenic adults. While contrary to work showing a positive relationship between total body creatine pools and muscle volume (Clark et al., 2014), our findings are in agreement with those by Möller et al that showed, when total creatine pools were separated by its constituents (PCr and creatine), muscle creatine levels were elevated in comparison to younger counterparts while PCr was reduced (Möller et al., 1980). The combined results of lower PCr with elevated muscle creatine levels may reflect an inability to replenish PCr levels at rest, thus reducing a readily available energy source during short, high‐intensity periods of contractile work (i.e., fall prevention). Specifically, creatine diffuses to the inner mitochondrial membrane space which allows the mitochondrial‐specific isoform of creatine kinase (MtCK) to transfer a phosphate to generate PCr. The MtCK‐dependent shuttling system, which provides nearly 80% of the energy transfer from the mitochondrial matrix to the cytosol (Aliev et al., 2011), is an integral source of energy during short, intense bouts of muscle contractile work. This is reflected in our correlative analyses which revealed lower in vivo PCr content is strongly associated with reduced peak power. Collectively, lower levels of PCr and elevated creatine in older sarcopenic muscle are in line with a prevailing paradigm that impaired energetics contributes to loss of mobility in aging (Schrack, Simonsick, & Ferrucci, 2010).

### 3.2 Potential role of PDE2 and membrane integrity on muscle size and function

Loss of membrane integrity is a hallmark of aging skeletal muscle in preclinical rodent models (Demontis, Piccirillo, Goldberg, & Perrimon, 2013) and appears to stem from various factors, including neuromuscular junction instability (Rowan et al., 2012) and loss of force transfer proteins (Hughes et al., 2016). The loss of membrane integrity has been suggested to contribute to the sarcopenic phenotype, as preclinical rodent models of aging have shown that membrane instability reduces muscle fiber size and increases the abundance of muscle fibers co‐expressing differing myosin heavy chain isoforms (Rowan et al., 2012). Elevated PDE content has been suggested to be an in vivo readout for cellular membrane damage (Burt, 1985), and previous cross‐sectional analyses have revealed an age‐induced increase in skeletal muscle PDE content (Satrústegui et al., 1988). In the current study, we further identified a singular PDE peak, PDE2, that is associated with the sarcopenic phenotype, as higher levels of PDE2 were observed in sarcopenic adults in comparison to age‐matched nonsarcopenic controls. Furthermore, PDE2 content was negatively associated with muscle volume and peak power of the quadriceps muscle in a group of older obese adults. Along with in vivo measures, biochemical analyses of muscle biospecimens revealed metabolites associated with membrane damage are elevated with sarcopenia. Specifically, GPE, a biochemical readout of PDE2, was increased in sarcopenic muscle and was associated with reduced muscle mass and function in older adults. While we did not examine membrane damage specifically, our findings suggest markers associated with membrane damage (PDE2 and GPE) are elevated in sarcopenic muscle and are related to lower muscle mass and function.

### 3.3 Skeletal muscle phospholipids and sarcopenia

Given the signatures indicating cellular membrane damage with sarcopenia, we next examined the principle skeletal muscle phospholipids, the major constituents of cellular membranes. We observed that older adults with sarcopenia had elevated levels of PC, PE, and PG in comparison to nonsarcopenic counterparts. Our findings are in line with observations by Baburina and Jackowski, who showed increases in GPE under conditions of elevated phospholipid synthesis in a cell culture model (Baburina & Jackowski, 1999). Aging has been shown to increase skeletal muscle phospholipids, as Schunk et al have shown, using ^31^P‐MRS, phosphomonoester content, an in vivo readout of PC and PE is elevated in muscle of older adults (Schunk et al., 1999). Additionally, recent evidence by Uchitomi and colleagues has shown differential phospholipid composition in aged rodent muscle, with the majority of phospholipids increased in aged skeletal muscle in comparison to younger counterparts (Uchitomi et al., 2019). The changes in phospholipid composition were associated with reduced type IIb myofiber size in aged muscle (Uchitomi et al., 2019), which is similar to our findings revealing a negative association between phospholipids (PC, PE, and PG) with muscle volume. While in contrast to recent reports that showed reducing PE levels in skeletal muscle by inhibiting key phospholipid synthesis pathways (phosphatidylserine decarboxylase) results in muscle atrophy (Heden et al., 2019; Selathurai et al., 2019), our findings and those by Uchitomi et al. (2019) reveal that age‐induced muscle atrophy may be linked to elevated phospholipids levels in skeletal muscle.

Along with muscle mass, we observed that elevated total PC and PE were negatively associated with peak power of the quadriceps muscle. A possible cause for this relationship could be due to the phospholipid composition of intracellular organelles. In particular, changes in the phospholipid composition of the sarcoplasmic reticulum, which regulates intracellular calcium levels by the sarcoplasmic reticulum calcium ATPase (SERCA), and the mitochondria, which provides energy in the form of ATP to fuel contractile activity, result in reduced membrane fluidity, impaired membrane integrity, and increased propensity for peroxidation and damage, leading to impaired function (Cortie et al., 2015; Shaikh, Sullivan, Alleman, Brown, & Zeczycki, 2014). In isolated sarcoplasmic reticulum fractions, an increase in PC levels and the PC:PE ratio by genetic manipulation, either by inactivation of fatty acid synthase or knockout of the terminal enzyme in the Kennedy pathway of phospholipid synthesis, choline/ethanolamine phosphotransferase 1 (CEPT1), led to a decrease in SERCA activity, resulting in muscle weakness (Funai et al., 2013, 2016). At the level of the mitochondria, Shaikh et al. have shown that fusion of isolated mitochondria with phospholipids impaired activities of complex I, II, and IV of the electron transport chain (Shaikh et al., 2014). While we observed an increase in the major phospholipids in skeletal muscle, we cannot discern the compartmentalization of these lipids, and whether changes in phospholipid composition of organelles led to the sarcopenic phenotype. As both mitochondria and SERCA appear to be involved in the regulation of muscle mass and function, it is critical to understand whether changes in lipid composition within these organelles are a potential mechanism for sarcopenia in older adults.

### 3.4 Study limitations

Using ^31^P‐MRS, our results reveal distinct in vivo phosphorus metabolites that pertain to lower skeletal muscle mass and function in older adults. To determine resting concentrations of in vivo phosphorus metabolites, ratios taken relative to a constant standard have been classically employed, including a constant concentration of βATP (5.5 mmol/kg; Wu et al., 2012) or PCr (27 mM; Conley et al., 2000). In the current study, we used the assumption that concentration of γATP is 8.2 mM which was based on a study of 81 muscle biopsy samples of young healthy adults (Harris et al., 1974). We acknowledge that further work validating these previous findings of metabolite concentrations is needed to provide an accurate assessment of phosphorus metabolite content in skeletal muscle using ^31^P‐MRS. While we observed significant relationships between PCr/PDE2 and markers of muscle mass and function, future work with a larger cohort of participants that vary in age and disease state is needed to explore the relationship between in vivo phosphorus metabolites and clinical characteristics pertinent to the sarcopenic phenotype.

## 4 CONCLUSIONS

In summary, ^31^P‐MRS analysis reveals distinct phosphorus metabolites (i.e., PCr and PDE2) that are altered sarcopenic muscle and are associated with muscle dysfunction. The changes in PCr were associated with increase muscle creatine levels, reflecting a potential impairment in phosphate shuttling between the mitochondria and cytosol. The differences in PDE2 may influence membrane integrity due to changes in skeletal muscle phospholipid composition. Whether the changes in resting in vivo metabolites are a causal factor in mediating the loss of muscle mass and function or just a consequence of the sarcopenic phenotype is unknown and requires future investigation. Regardless, the relationship between in vivo metabolites and markers of skeletal muscle mass, strength, and functionality point to an important influence of PCr and PDE2 on the sarcopenic phenotype, thus reflecting a potential therapeutic target to prevent or alleviate the detrimental effects of sarcopenia in the older adult population.

## 5 EXPERIMENTAL PROCEDURES

### 5.1 Participant recruitment

Seventy‐nine older men and women were recruited from the Orlando, FL area. The data reported here are baseline assessments of individuals enrolled in two separate clinical studies. In the first study, older adults with obesity (BMI > 30 kg/m^2^, *n* = 55; 12 males, 43 females) were enrolled (Clinicaltrials.gov identifier NCT02230839). The study consisted of individuals with (*n* = 18) and without (*n* = 37) type 2 diabetes. In the second study, enrolled older adults (*n* = 24; 12 males, 12 females) were classified as nonsarcopenic or sarcopenic based on the consensus definition of the EWGSOP (Cruz‐Jentoft et al., 2010). Beside diabetes diagnosis in a subset of *study 1* individuals, participants in both studies were in good general health, defined as weight stable for the last 6 months, and normal resting blood pressure (<150 mmHg systolic, <90 mmHg diastolic). Participants provided written informed consent before completing any data collection procedures. All experimental procedures were reviewed and approved by the AdventHealth Orlando institutional review board and were performed in accordance with the standards set forth by the Declaration of Helsinki.

### 5.2 Skeletal muscle function

Quadriceps contractile performance of the dominant leg was assessed using an isokinetic dynamometer (Biodex Medical Systems, Inc.). One repetition knee extension maximum (1‐RM) was determined by a standard weight stack. Weight was continually increased until test termination, with 5 min rest between attempts. For *study 2*, hand grip strength was tested using a Jamar^®^ hand‐held digital dynamometer. Each participant was given three attempts, and the average of these attempts was used for analysis.

### 5.3 Body and muscle composition

Body composition was assessed by dual‐energy X‐ray absorptiometry (DXA) using a GE lunar iDXA whole‐body scanner. Body composition was analyzed with encore software. Additionally, thigh muscle composition was determined by MRI using a 3T Philips Achieva magnet as previously described (Distefano et al., 2018). Muscle volume was determined by Analyze 11.0 software (Biomedical Imaging Resource, Mayo Clinic).

### 5.4 Magnetic resonance spectroscopy

Measurements were performed using a 3T Philips Achieva magnet as previously described (Distefano et al., 2018). Briefly, a 7 cm ^31^P surface coil was placed on the thigh to measure phosphorus metabolites using an unlocalized one‐pulse acquisition with block RF pulses. To improve spectral resolution to quantify smaller peaks within the phosphorous spectra, the fully relaxed spectrum was obtained with the same repetition time as previous studies (15 s; Szendroedi et al., 2011), but was repeated 48 times for a total acquisition time of 12 min. The areas/heights of each peak were determined using the advanced method for accurate, robust, and efficient spectral (AMARES) fitting algorithm within the java magnetic resonance user interface (jMRUI) software. Peaks were taken relative to the γATP peak, and steady state concentrations were calculated by setting the area under the γATP peaks equal to 8.2 mM (Harris et al., 1974).

### 5.5 Percutaneous muscle biopsies

Percutaneous muscle biopsies were performed in the morning after an overnight fast in *Study 2* participants. Participants were instructed to refrain from physical exercise 48 hr prior to the biopsy procedure. Participants rested supine for ~30 min prior to the biopsy. Biopsies were obtained from the middle region of the vastus lateralis (10–15 cm above the knee) under local anesthesia (2% buffered lidocaine) with a 5‐mm Bergstrom needle under suction. Following the biopsy, excess blood, visible fat, and connective tissue were removed from the specimen. About 50 mg of muscle tissue was immediately frozen in liquid nitrogen (−190°C) and stored at −80°C until metabolite and lipid analysis.

### 5.6 Lipidomics

Lipids were extracted from homogenized muscle samples with chloroform:methanol (1:1, by volume) as previously described (Kiebish et al., 2010). Lipidomic analysis was carried out on diluted samples (50× in isopropanol:methanol:acetonitrile:H_2_O (3:3:3:1, by volume) with 2 mM ammonium acetate) using an Ekspert MicroLC 200 system. The parameters of the mass spectrometer were optimized, and the samples were analyzed automatically using a data‐independent analysis strategy, allowing for MS/MSALL high resolution and high mass accuracy. Total number of lipids examined for each phospholipid species are as followed: PC, 108 (range: 108); PE, 79 (range: 70–91); PG, 157 (range: 129–231).

### 5.7 Metabolomics

Metabolomic analyses were performed using nontargeted and targeted protocols as previously described (Tolstikov, Nikolayev, Dong, Zhao, & Kuo, 2014). Metabolite extraction was achieved using a mixture of isopropanol:acetonitrile:water (3:3:2 v/v). Extract analysis was performed using gas chromatography–mass spectrometry, reversed‐phase LC‐MS, and hydrophilic interaction chromatography–liquid chromatography–tandem mass spectrometry protocol. A quality control was performed using metabolite standards mixture and pooled samples. Collected raw data were manually inspected, merged, and imputed.

### 5.8 Statistical analyses

Associations between skeletal muscle metabolites, mass, and function were examined by Pearson partial correlation analyses adjusted to gender. Multiple linear regression was used to determine independent cross‐sectional associations between PCr or PDE2 and peak power or muscle volume adjusted for T2D status, BMI, and fat mass. Analyses were performed using SPSS Statistics (Version 25). To assess the group differences, we applied a two‐way ANOVA with group, sex, and their interaction as factors in the models. If the interaction between group and gender was significant, post hoc comparison with stepdown Sidak correction was conducted to evaluate the differences between groups by sex. Data are presented as mean ± standard error (*SEM*). All analyses were performed in SAS (9.4), and statistical significance was set at *p* ≤ .05.

## CONFLICT OF INTEREST

P.M. Coen is a consultant for Astellas/Mitobridge, Incorporated.

## AUTHOR CONTRIBUTIONS

BHG and PMC contributed to study concept and design. JMH, HHC, RAS, EYC, NRN, BPG, VB, VVT, MAK, RBV, and PMC collected the data. JMH, HHC, FY, RBV, and PMC analyzed data. JMH drafted the manuscript. All authors reviewed and approved the manuscript. PMC is the guarantor of the data.

## Supporting information

Table S1

## Data Availability

The data that support the findings of this study are available from the corresponding author upon reasonable request.
