# Supplementary material for: Older adults with sarcopenia have distinct skeletal muscle phosphodiester, phosphocreatine, and phospholipid profiles
Source: Aging Cell. 2020 May 28;19(6):e13135. doi: 10.1111/acel.13135 (PMC7294783; doi:10.1111/acel.13135)
Supplement: Supplementary file 1 — Table S1 [file ACEL-19-e13135-s001.docx]

**SUPPORTING MATERIALS**

**Supplemental Table: Fat Composition Correlations**

|  | Fat Mass | | Region % Fat | |
| --- | --- | --- | --- | --- |
|  | Pearson r | P-value | Pearson r | P-value |
| Pi | -0.229 | 0.102 | -0.237 | 0.091 |
| PCr | -0.158 | 0.263 | -0.381 | 0.005* |
| PDE1 | 0.114 | 0.422 | 0.054 | 0.702 |
| PDE2 | 0.063 | 0.632 | 0.294 | 0.034* |
